# Acute lung injury caused by sepsis: how does it happen?

**DOI:** 10.3389/fmed.2023.1289194

**Published:** 2023-11-21

**Authors:** Baisheng Sun, Mingxing Lei, Jiaqi Zhang, Hongjun Kang, Hui Liu, Feihu Zhou

**Affiliations:** ^1^Department of Critical Care Medicine, The First Medical Centre, Chinese PLA General Hospital, Beijing, China; ^2^Medical School of Chinese PLA, Beijing, China; ^3^Department of Orthopedic Surgery, Hainan Hospital of Chinese PLA General Hospital, Beijing, China; ^4^Department of Orthopedic Surgery, National Clinical Research Center for Orthopedics, Sports Medicine and Rehabilitation, Beijing, China; ^5^Medical Engineering Laboratory of Chinese PLA General Hospital, Beijing, China

**Keywords:** sepsis, acute lung injury, pathogenesis, inflammation, treatment

## Abstract

Sepsis is a systemic inflammatory disease caused by severe infections that involves multiple systemic organs, among which the lung is the most susceptible, leaving patients highly vulnerable to acute lung injury (ALI). Refractory hypoxemia and respiratory distress are classic clinical symptoms of ALI caused by sepsis, which has a mortality rate of 40%. Despite the extensive research on the mechanisms of ALI caused by sepsis, the exact pathological process is not fully understood. This article reviews the research advances in the pathogenesis of ALI caused by sepsis by focusing on the treatment regimens adopted in clinical practice for the corresponding molecular mechanisms. This review can not only contribute to theories on the pathogenesis of ALI caused by sepsis, but also recommend new treatment strategies for related injuries.

## 1 Introduction

Sepsis is a systemic inflammatory response syndrome caused by bacteria, viruses and other pathogenic microorganisms in the body (1–3). It is also one of the most important causes of death of critically ill patients (4, 5). According to the version 3.0 Guidelines of International Consensus on Sepsis (Third Edition, 2016), sepsis is a multi-organ dysfunction caused by host inflammatory response disorders dominated by infections (6–8). The morbidity and mortality of sepsis remain high, moreover, the high hospitalization rate and the high mortality rate have pushed the health care cost of this disease to the top (8, 9). During the process of multiple organ dysfunction caused by sepsis, the lung is the earliest and most susceptible target organ (10, 11). Research indicates that 25 to 50% of sepsis patients experience acute lung injury (ALI), with a mortality rate of 40% (12, 13). Patients with sepsis-induced ALI (S-ALI) have weakened gas exchange function due to lung inflammation and tissue damage. Pathological processes include pulmonary vascular endothelial damage, reduced alveolar surface tension, inflammatory factor release and pulmonary interstitial fibrosis (14, 15). The clinical manifestations are systemic inflammatory response syndrome characterized by stubborn hypoxemia and respiratory distress (16, 17).

The occurrence and development of ALI caused by sepsis is a complex process involving multiple pathways and genes (18, 19). Previous studies have failed to elucidate the pathogenesis of this disease, and there are currently no effective treatment methods available. Therefore, exploring the pathophysiological mechanisms for and possible therapeutic interventions in ALI caused by sepsis is of great significance for clinical patients and prognosis. In this review, the research progress in pathogenesis of ALI caused by sepsis is summarized in terms of inflammatory cell mechanisms (20), oxidative stress mechanisms (21), coagulation system mechanisms (22), pulmonary surfactant (23) and genetic mechanisms (24). The feasible therapy strategies regarding the variety of induced molecular mechanisms are also outlined.

## 2 Definition and epidemiology of ALI caused by sepsis

ALI caused by sepsis is a severe inflammatory response of infection that causes damage to the alveolar wall and pulmonary exudation, leading to impaired lung function. The clinical manifestations of patients with ALI caused by sepsis include difficulty in breathing, hypoxemia, cough, fever, and accelerated heartbeats. Severe patients may experience shock and multiple organ dysfunction (25, 26). Severe sepsis with ALI can endanger a patient’s life. S-ALI is different from acute respiratory distress syndrome (ARDS) in that the latter is caused by multiple factors, while the former is caused by infections (27–29).

The Institute for Health Metrics and Evaluation (IHME) of the University of Washington conducted a statistical analysis of the morbidity and mortality of sepsis in the world between 1990 and 2017. The results showed that in 2017 about 48.9 million cases of sepsis were recorded globally, 11 million of which died due to sepsis, accounting for 19.7% of the global death toll (30). The global incidence of sepsis is as high as 437 cases/100000 person years, and sepsis accounts for 6% of the number of admissions in the United States. An epidemiological study on the prevalence, prognosis, and treatment of severe sepsis in children was conducted in 128 institutions from 26 countries worldwide for the first time. 6,925 patients were screened, and 569 developed severe sepsis (the prevalence rate was 8.2%, with a 95% confidence interval of 7.6–8.9%). The age of patients averaged 3.0 years old (the interquartile range was 0.7–11.0), and the hospital mortality rate was 25% (31). In 2017, an epidemiological survey based on population sepsis in China showed that the incidence of sepsis was 461/100000, and the case fatality rate was 79/100000 (32). In addition, another survey in China showed that the mortality rate of sepsis was 12.6%. An epidemiological survey of 2,322 sepsis patients from 44 ICUs showed that 68.2% of sepsis patients had ALI, with a 90-day mortality rate of 35.5% (33).

Reportedly, 150,000 to 200,000 patients worldwide die annually of S-ALI caused by sepsis (5). S-ALI is one of the main causes of death in ICUs. The mortality rate of S-ALI patients is reportedly significantly higher than that of non-sepsis ones, with the 60 day mortality rates of the two as high as 38.2 and 22.6%, respectively (34). In addition, patients with underlying diseases such as diabetes, cardiovascular diseases, pulmonary diseases, and liver dysfunctions are more vulnerable to S-ALI (35, 36), especially the elderly and patients with low immune function, whose mortality are higher (37). Because of its high morbidity and mortality, this disease has become one of the targets of medical research in recent years.

## 3 The pathogenesis of S-ALI

**Figure 1:**
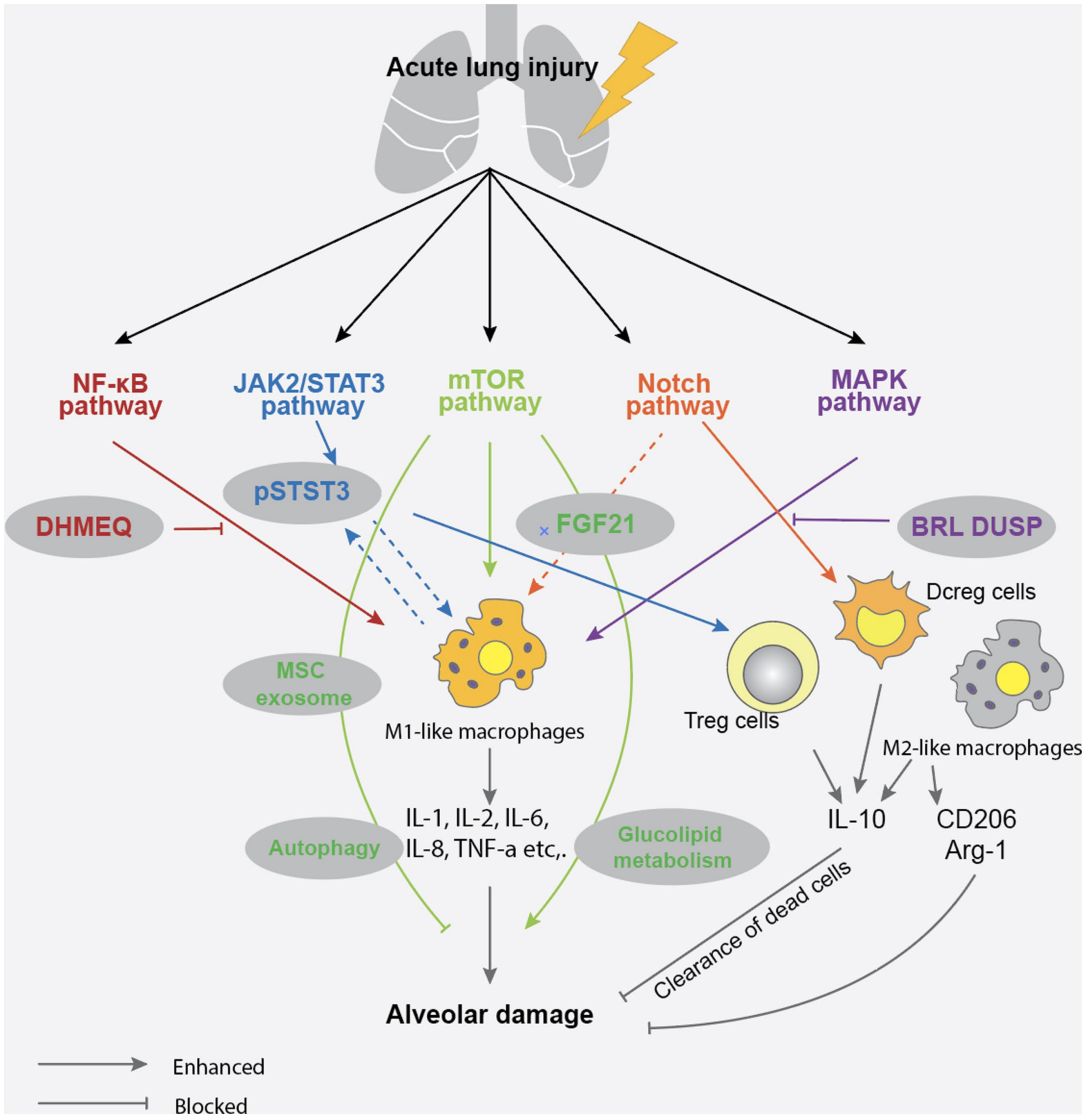
Acute lung injury in sepsis mainly causes alveolar damage through NF-KB, JAK2/STAT3, mTOR, Notch, and MAPK pathways, leading to impaired gas exchange function and inflammatory exudation.

The alveolar epithelial cells are composed of two types of cells: type I and type II. They combine with alveolar vascular endothelium to form a layer of alveolar wall capillary barrier, which can protect lung tissue from bacterial and viral infections under normal physiological conditions (38). Interestingly, type I alveolar epithelial cells exhibit plasticity during alveolar development and have a protective effect on lung barrier function (39). As a type of stem cell, type II alveolar epithelial cells can proliferate and differentiate into type I alveolar epithelial cells, and participate in the synthesis and secretion of surfactants that regulate alveolar surface tension. They play an essential role in enhancing alveolar fluid clearance and reducing pulmonary inflammatory response (40). Due to the protective effect of alveolar epithelial cells on the lung tissue barrier, the onset of ALI can manifest as apoptosis of alveolar epithelial cells and damage to the alveolar wall capillary barrier (41, 42). The mechanisms by which sepsis leads to the development of ALI are shown in Figure 1.

Regarding the mechanism of apoptosis of alveolar epithelial cells and damage to the alveolar wall capillary barrier, treatment measures include respiratory support therapy, lung protective ventilation, nutritional support and sufficient fluid management, use of antioxidants and anti-inflammatory drugs and immunotherapy (43–46). It should be noted that due to the complex pathogenesis of ALI caused by sepsis, alveolar epithelial cell apoptosis and alveolar wall capillary barrier damage require individualized treatment.

### 3.1 Inflammatory response mediated injury

#### 3.1.1 The impact of monocytes and macrophages on the process of ALI

**Figure 2:**
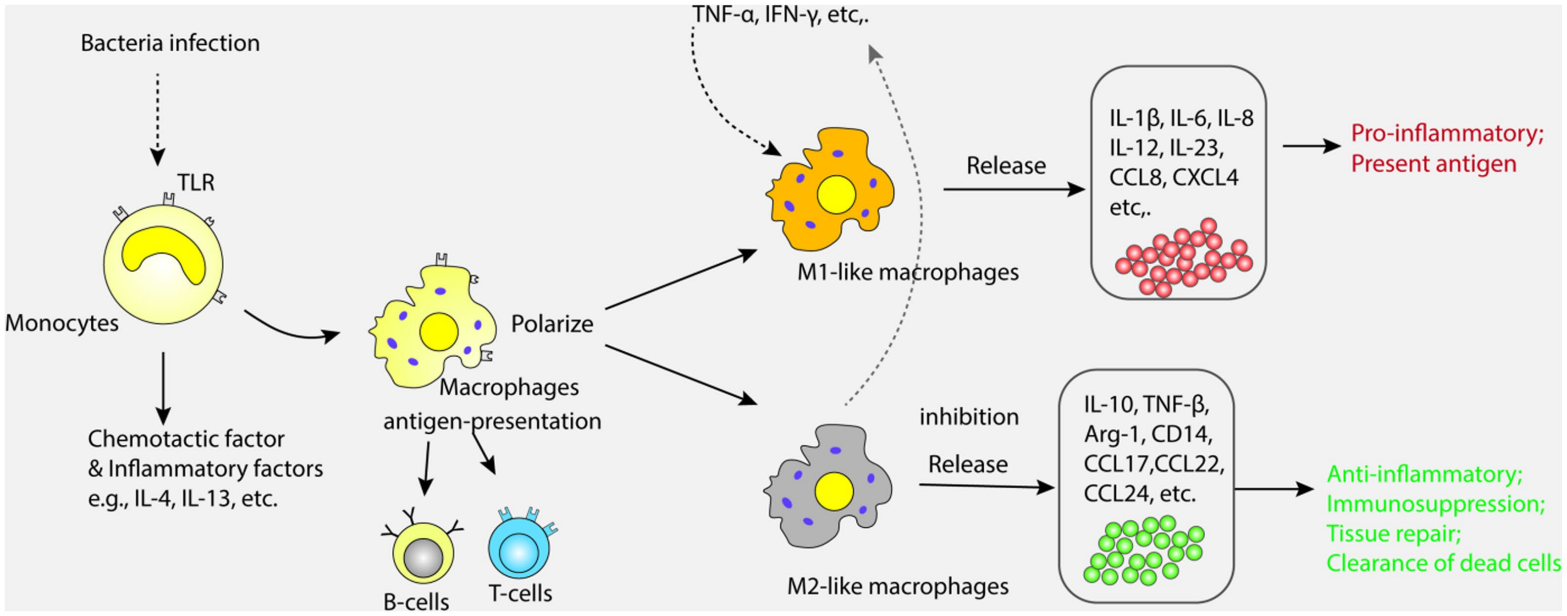
Monocytes recognize and bind microbial components through Toll-like receptors (TLR) on their surface, releasing a variety of inflammatory mediators and chemokines. Monocytes can also differentiate into macrophages, which are polarized into classical macrophages (M1) and alternative macrophages (M2). M1 and M2 promote the release of pro-inflammatory and anti-inflammatory factors, respectively, causing inflammatory response and injury in the lung.

Monocytes are mainly distributed in bone marrow and blood, and are important cells for innate immune response (47, 48). Monocytes can recognize and bind microbial components through the toll like receptor (TLR) on the surface, release a variety of inflammatory mediators and chemokines, and stimulate the further development of inflammatory response (49, 50). In addition, monocytes can also differentiate into macrophages and affect the function of other immune cells through the synergistic effect of cytokines released (51). In cases of inflammatory injuries in the body, monocytes can migrate to the infection site of inflammatory reaction and differentiate into residential tissue macrophages that contain multiple receptors on their surfaces and produce different polarization states under the stimulation of different microorganisms (52, 53). Among them, those polarized with pro-inflammatory phenotype are called classical macrophages (M1), while those polarized with anti-inflammatory phenotype are called substitute macrophages (M2) (54). Under the induction of cytokines which can be detected in tumor necrosis factor-α (TNF-α) and interferon-γ (IFN-γ), the release of pro-inflammatory cytokines such as interleukin-1 (IL-1), IL-6, and IL-8 in M1 exacerbates the body’s inflammatory response. M2 can inhibit the release of pro-inflammatory factors and promote the secretion of IL-10 and transforming growth factor-β (TGF-β), anti-inflammatory cytokines and effectively eliminate apoptotic cells. Research has shown that serum macrophage inhibitory factor (MIF), an important regulator of innate immunity, is closely related to ALI and the severity of the disease in sepsis patients (55, 56). Bacterial antigens stimulate white blood cells to release MIF, which binds to CD74 in other immune cells and triggers an acute immune response. Overexpressions of MIF can lead to excessive activation of inflammatory responses, leading to lung injury and organ dysfunction (57). Inhibiting MIF can alleviate the severity of ALI caused by sepsis, reduce inflammatory response and cell apoptosis (58). In fact, macrophages are one of the most important cell types in the process of ALI caused by sepsis. When pathogens invade the lungs, macrophages can recognize and ingest microorganisms such as bacteria, and activate T and B cells through antigen presentation, thereby inducing and enhancing the body’s immune response (59). In the process of macrophage activation, the TLRs on its surface binds to the antigen, which can activate NF-κB signal and MAPK pathways lead to the release of various inflammatory mediators and chemokines, attract a large number of inflammatory and immune cells into the lungs, and cause lung inflammatory response and damage (60, 61). Research has shown that various types of TLRs, including TLR2, TLR4, TLR5, TLR9, are involved in the pathogenesis of ALI caused by sepsis (62, 63). Therefore, inhibiting the TLR signaling pathway can alleviate the inflammatory response and severity of ALI caused by sepsis. For example, treatments targeting at TLR4 involve the use of TLR4 antibodies, TLR4 antagonists, and TLR4 signal transduction inhibitors (62). The treatment methods for TLR9 include the use of TLR9 antagonists (63). However, it should be noticed that since TLRs play an important role in the immune response of the body, inhibiting the TLR signaling pathway may affect the body’s immune defense ability, hence we need assess the safety and effectiveness of treatments. Also, macrophages can participate in the repair and regeneration process of lung injury by phagocytosing dead and fragmented cells, clearing inflammatory mediators, and secreting cell growth factors (64). The role and mechanism of mononuclear-macrophages in ALI are shown in Figure 2.

In summary, monocyte-macrophages play an important role in the pathogenesis of ALI caused by sepsis, and their abnormal activation and dysfunction can lead to excessive inflammatory response and extensive damage to lung tissue. The current treatment strategies for this mechanism are also a hot spot. It is found that inflammatory cytokines such as IL-1β and TNF- α can alleviate inflammatory reactions and tissue damage (42, 65). Anti-cytokine drugs such as tocilizumab, adalimumab and celastrol have been used clinically to treat ALI (66–68).

#### 3.1.2 The impact of neutrophils and neutrophil extracellular traps on the ALI process

Neutrophils are briefly surviving granulocytes that are the initial defense against invading pathogens. They recruit other immune cells through phagocytosis, degranulation, and production of reactive oxygen species, chemokines, and cytokines to maximize the host’s immune response, thus achieving this goal (69). Abnormal activation of neutrophils is one of the hallmarks of ALI (70). In the process of ALI, neutrophils accumulate in the lungs, increasing the expression of inflammatory cytokines, damaging epithelial and endothelial integrity, expanding the alveolar arterial oxygen gradient and promoting the development of interstitial pulmonary edema. It is believed that a large number of neutrophils produced during the pathogenesis of S-ALI are mainly mediated by death. When the body gradually recovers, apoptosis is the main pathway for neutrophils. Studies by Lea F. et al. have shown that neutrophil apoptosis is inversely proportional to the severity of sepsis (71). Neutrophils enhance their antibacterial properties by releasing extracellular chromatin modified by histones and neutrophil extracellular traps (NETs) composed of many granular proteins (72), and are identified as part of the innate immune response, which may be beneficial or pathological. NETs have been a research hotspot in recent years. NETs components include histones, cathepsin G, neutrophil elastase (NE), myeloperoxidase (MPO), lactoferrin, antimicrobial peptide-LL37, all of which have bactericidal effects (73). The formation of NETs activates neutrophils by recognizing stimuli and activating the NADPH oxidase (NOX) complex through protein kinase C (PKC) - Raf/MERK/ERK, thereby activating myeloperoxidase (MPO), neutrophil elastase (NE), and arginine deaminase type 4 (74). Studies have shown that excessive NETs play a pathological role in diseases, infectious and not, but not limited to thrombosis, diabetes, vasculitis or cancer (75, 76). With the exploration of research, an increasing number of scholars have found that NETs may play a negative role in sepsis by promoting the occurrence and development of inflammation. A controlled experiment in acute respiratory distress syndrome (ARDS) patients with pneumonia or sepsis found that an increase in plasma NETs levels and the severity of ARDS were associated with an increase in mortality through ELISA technology detection. Furthermore, the use of DNase I treatment in mouse models of severe bacterial pneumonia and acute lung injury reduced NETs and lung injury (77). A small cohort study of patients with septic shock in the intensive care unit found that an increase in levels of circulating NETs biomarkers (free DNA/myeloperoxidase complexes) was associated with the severity of organ dysfunction and 28 day mortality in patients with septic shock (78). Zhu S et al. found that NETs induced endothelial cell damage and produce a large number of tissue factors by stimulating the activation of interferon genes (STING). Amplifying the dysregulation between inflammation and coagulation leads to poor prognosis in S-ALI model mice, and disruption of NETs and inhibition of STING reduces inflammation and coagulation, thereby improving prognosis in sepsis mice (79). It can be seen that the increase in the level of NETs is related to the aggravation of S-ALI, and the mechanism of NETs in intravascular coagulation and intestinal inflammation in sepsis has attracted the most attention.

#### 3.1.3 The role of inflammatory cytokines in the ALI process

Inflammatory cells in the human body release a variety of cytokines, including pro-inflammatory cytokines that can cause inflammatory reactions and anti-inflammatory cytokines that can fight inflammatory reactions (80). The balance between pro-inflammatory cytokines and anti-inflammatory cytokines plays an important role in maintaining the stability of the internal environment. In case of imbalance, uncontrolled systemic inflammatory response syndrome (SIRS) will occur. The onset of SIRS is essential in the pathogenesis of ALI caused by sepsis (81). The pro-inflammatory cytokines and inflammatory mediators in the process of sepsis mainly include tumor necrosis factor-α (TNF-α), interleukin-1β, IL-2, IL-6, IL-8, platelet activating factor (PAF) and phospholipase A2 (82, 83). Among them, TNF-α is an important promoter of sepsis and sepsis associated ALI, inducing the production of pro-inflammatory factors such as IL-6 and IL-8 and is an important systemic reactive mediator (84). In addition, studies had shown that high levels of IL-1β in the body were associated with sepsis, the IL-1β levels in the dead were higher than that of survivors (85).

It is worth noticing that high levels of IL-6 can activate the coagulation system, increase vascular permeability, and provide conditions for the rapid spread of inflammation (86). High levels of IL-6 can trigger pro-inflammatory IL-6 mediated signaling cascades, and IL-6 binding to soluble interleukin-6 receptors (sIL-6R), which can bind to gp130 of membrane proteins, consecutively activating the JAK–STAT pathway (87). In addition, due to the widespread expression of gp130 in many effector cells, high levels of IL-6 can lead to stronger immune activations (88). Research had found that the average level of IL-6 in patients with severe sepsis was significantly higher than that in healthy individuals (<10 pg./mL), which is tens or even hundreds of times higher than that of normal individuals (89). Moreover, the release of platelet activating factor (PAF) is closely related to the formation of lung injury caused by sepsis. PAF is a type of lipid immune mediator that can cause platelet aggregation reactions. Neutrophils, mast cells, macrophages and monocytes can release PAF under the stimulation of specific antigens and endotoxin (90). PAF can cause symptoms such as constriction of the bronchus, airway hyperresponsiveness, and pathophysiological changes such as pulmonary edema and pulmonary hypertension, which further aggravate lung injury.

The onset of ALI caused by sepsis is related to the activation of complex inflammatory cytokine cascades. Various pro-inflammatory cytokines and inflammatory mediators interact and influence each other, promoting the development of lung injury. Treatment measures based on cytokine mechanism include glucocorticoid, cytokine interceptor and phosphodiesterase-4 inhibitor (91–94). In addition, alkaloids, intratracheal injection of epidermal growth factor, and alveolar lavage also have therapeutic effects, but more research is needed to confirm their effectiveness and safety.

### 3.2 The role of oxidative stress mechanism in ALI process

In ALI caused by sepsis, oxidative stress mechanisms also play an important role. Oxygen free radicals have physiological effects such as killing microorganisms, clearing necrotic or aging cells, and regulating inflammatory responses. Under normal physiological conditions, the oxygen free radicals produced in the lungs can be maintained in a balanced state of production and clearance, but excessive release or decreased clearance ability of oxygen free radicals can cause damage to lung tissue cells (95, 96). And microorganisms invade lung tissue, phagocytes and endothelial cells release a large number of inflammatory factors (97), which can activate effector cells such as alveolar macrophages and multinucleated leukocytes, release a large number of oxygen free radicals and (be reduced to O_2_^−^, most O_2_^−^ is disproportionated to H_2_O_2_ under the action of superoxide dismutase), H_2_O_2_ combines with O_2_^−^ to form OH, and myeloperoxidase can oxidize H_2_O_2_ to produce hypochlorous acid and other toxic substances, it directly damages the alveolar epithelial cells and vascular endothelial cells, affects the gas exchange of the alveoli, and eventually leads to serious lung injury and pulmonary function decline (98). Zou et al. found that the use of oxygen free radical scavenger can protect lung tissue in the rat model of lipopolysaccharide induced ALI (99). Reduced expressions of NF-κB and TNF-α reveals a good inhibitory effect on pulmonary inflammatory response. Therefore, treatment strategies for oxidative stress have become one of the research hotspots.

**Figure 3:**
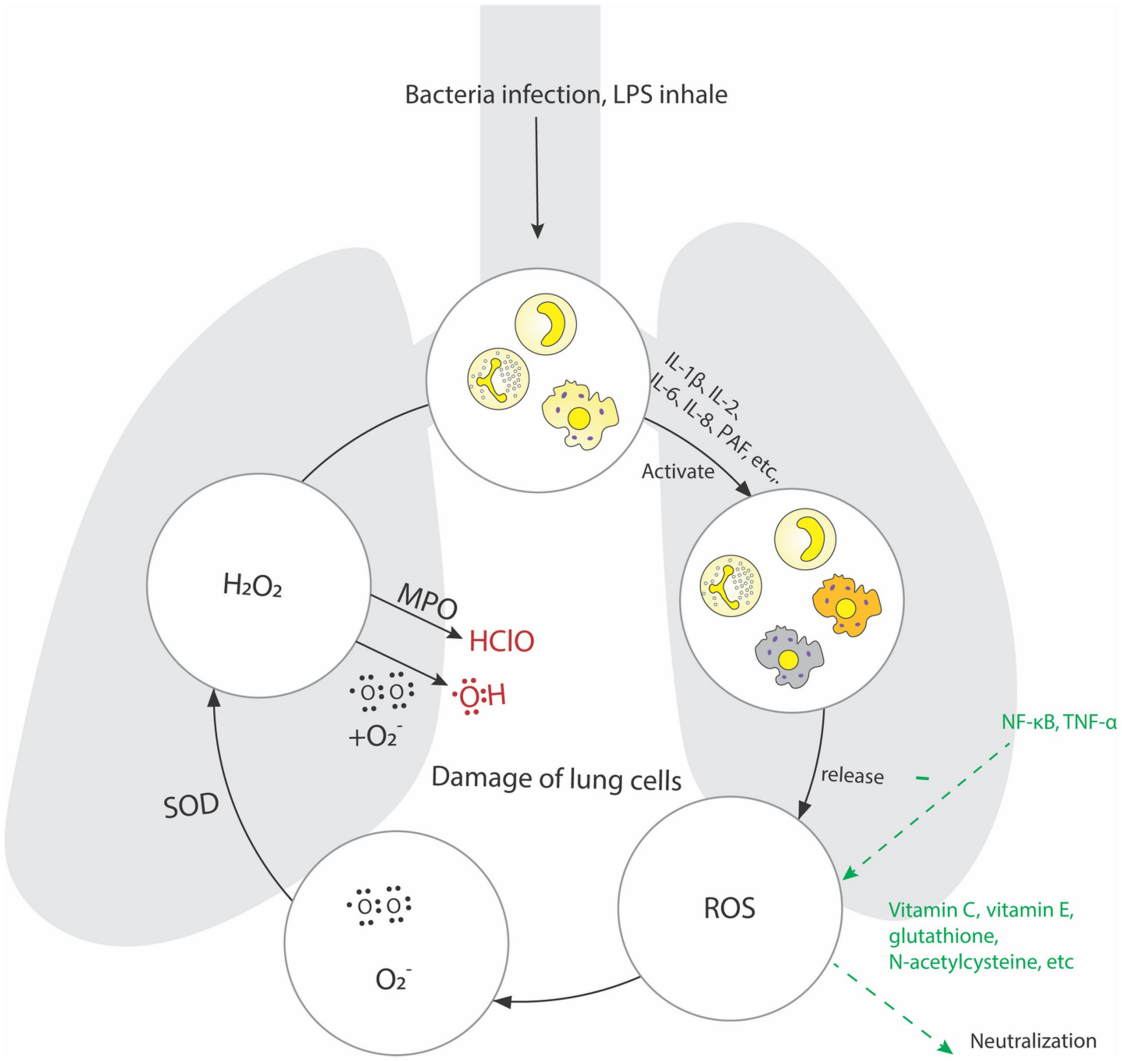
During sepsis, phagocytes and endothelial cells in the lung tissue release a large number of inflammatory factors, which activate alveolar macrophages, multinucleated leukocytes and other effector cells. Release a large number of oxygen free radicals and be reduced to O_2_^−^, most O_2_^−^ is disproportionated to H_2_O_2_ under the action of Superoxide dismutase, H_2_O_2_ combines with O_2_^−^ to form OH, and Myeloperoxidase can oxidize H_2_O_2_ to produce hypochlorous acid and other toxic substances, it directly damages the alveolar epithelial cells and vascular endothelial cells.

Under normal circumstances, the body constantly generates oxygen free radicals (OFR) in metabolic reaction and enzyme catalysis. However, because of the existence of the OFR enzyme system in the body, OFR can be cleared to achieve balance. Excessive production of reactive oxygen species (ROS) in the body or a decrease in enzymes that clear OFR can affect lipid, protein, and nucleic acid metabolism, leading to an imbalance between the oxidation and antioxidant systems and damaging the body. Research has shown that excessive ROS can cause ALI, leading to cell damage, activating pro-apoptotic signaling pathways, and ultimately leading to the death of alveolar epithelial and endothelial cells (96). Some researchers have also found that excessive production of ROS or reduced antioxidant capacity can further increase the level of oxidized phospholipids, which are closely related to lung injury, lung infection, and cell apoptosis (97). In addition, some scholars have found that excessive ROS may also activate NF-κB, thus inducing ALI (98). It is precisely because of the recognition of the role of oxidative stress response in the pathogenesis of ALI that researchers are committed to developing drugs such as perfluorocarbons (PFCs) that reduce ROS production or enhance antioxidant capacity as clinical treatments for ALI (99). The mechanisms of action of oxidative stress in the process of ALI are summarized in Figure 3.

After lung injury occurs, edema, ischemia and hypoxia can lead to mitochondrial dysfunction in cells, producing a large number of oxygen free radicals and generating active oxygen. The ability of the body to scavenge free radicals is limited. A large amount of active oxygen in the lung tissue is an important substance for the enhancement of pulmonary vascular permeability and cell damage, which ultimately causes pulmonary edema and lung injury (100). The clinical treatment strategies for antioxidant stress are currently N-acetylcysteine, ambroxol and some Chinese herbal ingredients, such as xiyanping, ferula and *lycium barbarum*. As the main drug for scavenging oxygen free radicals, ambroxol can avoid oxidative damage to cell lipids, thus inhibit TNF-α、IL-6, which can improve the expression of superoxide dismutase in patients’ serum and effectively alleviate hypoxemia. Some natural or synthetic antioxidants, such as vitamin C and vitamin E, glutathione and N-acetylcysteine, can neutralize free radicals, reduce oxidative stress and tissue damage (100–103). In addition, substances and enzymes that regulate the redox state are also important pathways for treating oxidative stress (104, 105).

### 3.3 The role of coagulation system mechanisms in the ALI process

**Figure 4:**
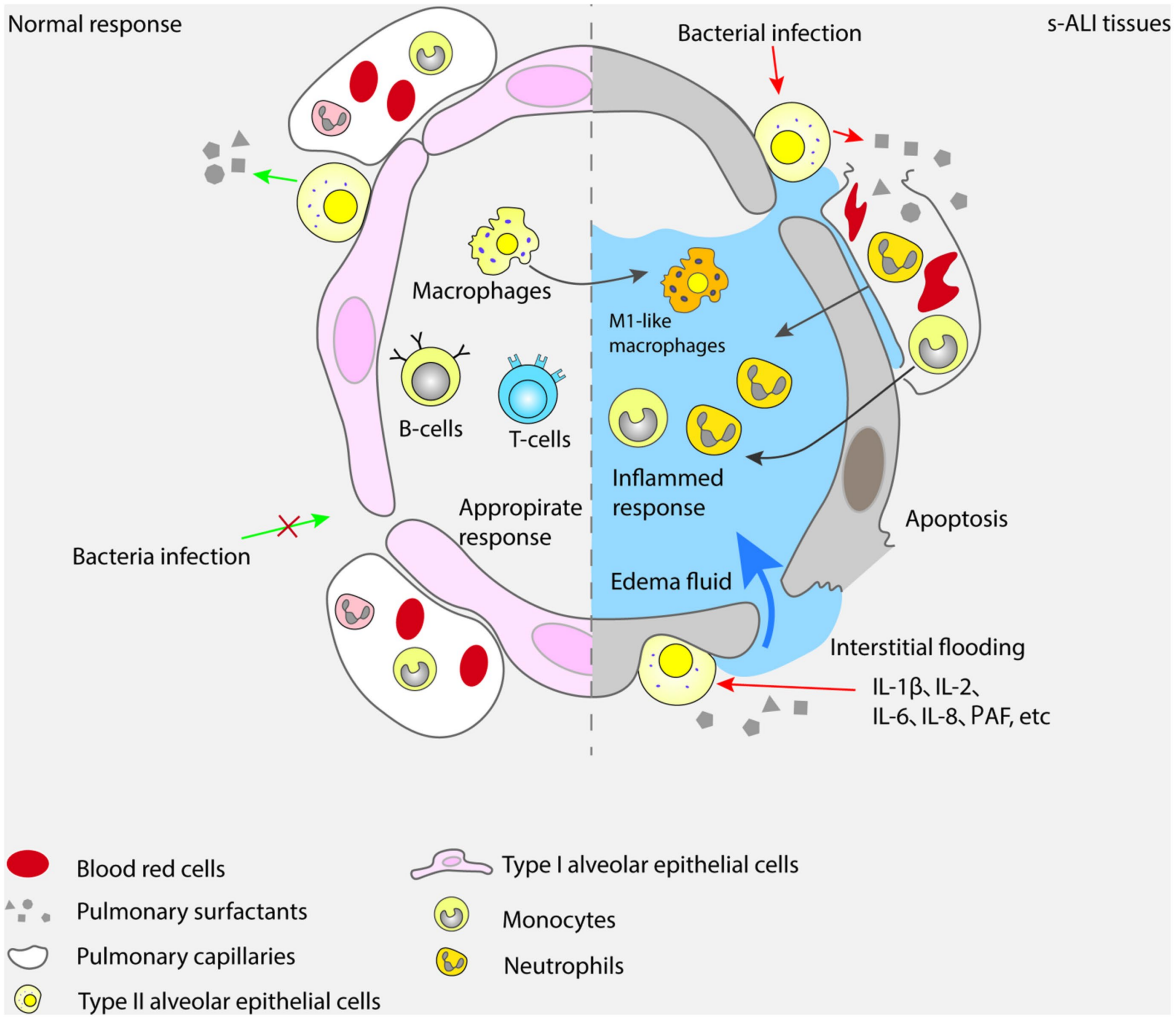
In sepsis, the coagulation system is activated, the damage of vascular endothelial cells leads to thrombosis and fibrin deposition, and the disorder of anticoagulant system, which aggravates lung injury and dysfunction.

In ALI caused by sepsis, the coagulation system also plays an important role. Along with inflammatory reactions and tissue damage, the coagulation system is also activated, leading to thrombosis and deposition of fibrin. Inflammatory reactions and tissue damage can lead to damage and activation of vascular endothelial cells, exposing coagulation factors on the surface of vascular endothelial cells. At the same time, inflammatory cells also release pro-coagulant substances such as tissue factors and platelet activating factors, promoting platelet aggregation and thrombosis. ALI caused by sepsis will also lead to the imbalance of the anticoagulant system, which will increase the level of plasmin activator inhibitor (PAI-1) and decrease the level of antithrombin (AT). This imbalance will further promote thrombosis and fibrin deposition. Clinically, sepsis patients generally have abnormal exogenous coagulation pathways, exotoxin, endotoxin and various cytokines that can promote monocytes and endothelial cells to release tissue factor (TF) in large quantities, thus activating exogenous coagulation pathways and secreting thrombin (106). TNF-α inflammatory cytokines which also promote the inhibitor of plasmin activator. Under the combined action, the coagulation level increases and the fibrinolysis system is inhibited (107). Graf et al. reported that coagulation abnormalities commonly associated with infection and inflammatory reactions were due to tissue factor mediated thrombin production (108). With fibrinolysis and promotion of fiber proliferation, systemic inflammatory reactions lead to activation of the coagulation system, which can directly or indirectly affect the inflammatory response. In the pathological process of ALI caused by sepsis, dysfunction of the coagulation system can lead to thrombosis and fibrinogen deposition, exacerbating lung injury and dysfunction. Studies have shown that the use of anticoagulants such as tissue factor pathway inhibitor, antithrombin, heparin, activated protein C and plasmin activator, especially tissue type plasmin activator, can significantly improve lung function and increase oxygen supply in ALI and ARDS (109, 110). The mechanism of action of the coagulation system in ALI caused by sepsis is illustrated in Figure 4.

Platelets play an important role in the process of lung injury. Nonsteroidal anti-inflammatory drugs play an anti-inflammatory role by inhibiting platelet aggregation. Aspirin should be used for primary and secondary prevention, achieving lung injury inhibition through inhibiting platelet aggregation and regulating immune function. Aspirin is used during the period of pathological change of lung tissue after paraquat poisoning. It can be converted into AT-RvD1 in the airway mucosa under the catalysis of acetylated cyclooxygenase-2, thus reducing the content of TNF- α and IL-1 β in lung tissue caused by paraquat. At the same time, aspirin can also reduce the degree of lung tissue damage and lung injury score by triggering lipoprotein A4, thereby reducing the concentration of white blood cells and proteins in bronchoalveolar lavage fluid (111). At the same time, the application of antiplatelet therapy in ALI patients can help reduce the levels of serum D-dimer, NT-pro BNP and platelets, promote the reduction of lung injury and the risk of ARDS. The current treatment strategy for the coagulation system is also one of the hot spots in the research and treatment of ALI caused by sepsis. The treatment methods include the use of anticoagulant, fiber solvents and platelet inhibitors (111, 112).

### 3.4 Role of pulmonary surfactant in ALI

Pulmonary surfactant (PS) is a complex mixture synthesized of lipids and specific surfactant binding proteins secreted by alveolar type II epithelial cells. PS forms a lining layer between the respiratory lining fluid and the inhaled air (113, 114). Pulmonary surfactant mainly exists on the surface of the alveoli, which can regulate the surface tension of the alveoli and prevent the collapse of the alveoli, and maintain the stability and elasticity of the lungs (113, 115, 116). In ALI caused by sepsis, inflammatory reaction and cytokine release will lead to lung injury and abnormal changes of alveolar surfactant, and the alveolar surface tension will increase, leading to alveolar collapse and ventilation disorder. The lack or abnormality of pulmonary surfactant will also aggravate this pathological process (114). What’s more, the damage to alveolar capillaries and the increased permeability of capillaries can also lead to the infiltration of pulmonary surfactant into plasma, which is expressed at a low level in alveolar lavage fluid (117). The research of Czyzewski et al. showed that the lack or decrease of pulmonary surfactant in the alveolar lavage fluid of patients with ALI would lead to more severe respiratory distress symptoms of patients (118).

The decrease in pulmonary surfactant content has the following reasons. Firstly, patients with ALI have varying degrees of type II alveolar epithelial cell damage and a large secretion of TNF-α and various interleukins, thereby inhibiting the synthesis of pulmonary surfactant. Secondly, the increase of protease activity in patients with ALI leads to increased degradation of pulmonary surfactant which is a lipoprotein complex. At the same time, the damage to alveolar capillaries and the increased permeability of capillaries can also lead to the infiltration of pulmonary surfactant into the plasma, leading to it is expression at a low level in alveolar lavage fluid (119). Pulmonary surfactant proteins have the function of regulating local immunity and inflammatory responses. Pulmonary surfactant protein A (SP-A) and pulmonary surfactant protein D (SP-D) can attenuate lipopolysaccharide induced apoptosis and increase of caspase3 and BAX/Bcl-2 in intestinal epithelial cells (IEC), which confirms that SP-A and SP-D have protective effects on apoptosis (119). By means of supplementing pulmonary surfactant, lung function can be improved and inflammatory reaction can be alleviated (120, 121). However, further research is needed to determine the optimal therapeutic dose and time to give pulmonary surfactant.

### 3.5 The role of genetic mechanisms in the ALI process

Although the relationship between genetic inheritance and ALI caused by sepsis has not yet been fully established, it is obvious that predisposing genetic constitution contributes to the occurrence and severity of ALI caused by sepsis (122). Bime et al. said that the expression of selectin P ligand gene in the mouse lung injury model is related to ethnic groups, suggesting that gene differences may be the reason for the differences in the body’s responses to inflammatory response regulation (123). It is believed that ALI caused by sepsis is a multipathway and multi gene regulatory process, in which noncoding RNA (ncRNA) plays an important role. NcRNA is a type of RNA that cannot encode proteins, but can regulate gene expressions at the genomic and chromosomal levels, determine cell differentiation fate, and participate in the occurrence and development of various diseases (124). MicroRNAs (miRNAs) are a type of noncoding RNA encoded by endogenous genes with a length of approximately 22 nucleotides, which inhibits the expression of target genes by directly binding to their mRNA. Research has shown that microRNAs participate in the occurrence and development of ALI caused by sepsis through mediating the release of inflammatory factors, expression of pulmonary vascular endothelial related proteins, and cell apoptosis which can affect downstream targets (125, 126). Claudia et al. pointed out that inhibiting microRNA-193b-5p (miRNA-193b-5p) *in vivo* can alleviate ALI caused by sepsis (124). Jiang et al. found through *in vivo* experiments that intravenous injection of serum extracellular vesicles obtained from ALI mice can increase the number of M1 macrophages in the lungs of young mice and cause lung inflammation (127). The serum extracellular vesicles of ALI mice transmit miR-155 to macrophages, stimulating nuclear factors κB (NF-κB) activation, resulting in the production of tumor necrosis factor α (TNF-α) and IL-6. Research has found that miR-155 released from serum extracellular vesicles promotes macrophage proliferation and inflammation by targeting SHIP1 and SOCS1, respectively; suggesting that the miR-155/SHIP1/SOCS1 axis of serum extracellular vesicles was one of the pathogeneses of ALI caused by sepsis, and miR-155 may become a potential target for preventing and treating ALI. In addition to the association between microRNAs and ALI caused by sepsis, research has found that the levels of long chain noncoding RNAs (lncRNAs) are significantly elevated in the plasma of patients with ALI caused by sepsis, and can predict the risk of ALI caused by sepsis and evaluate the condition and prognosis of patients (128). LncRNA plays an important role in LPS induced pulmonary endothelial inflammation and barrier dysfunction, which further confirms the role of lncRNA in the pathogenesis of acute ALI caused by sepsis (53). Unfortunately, there is not any finding on the application of lncRNA in ALI targeted therapy so far. Wang et al. found that lncRNA may be a potential preventive and therapeutic target for ALI by using techniques such as microarray analysis, bioinformatics analysis, and real-time quantitative PCR (129). Research on the expression change of circular RNA in human umbilical vein endothelial cell (HUVECs) caused by hypoxia, the impact of circular RNA on proliferation, migration and apoptosis of HUVECs showed that circular RNA played a role in the endothelial barrier function, suggesting that it might play a role in the pathogenesis of ALI caused by sepsis (130). Scholars have studied the effect of circRNA knockout on LPS induced ICAM-1 expression, and found that knocking down the expression of circRasGEF1B can reduce LPS induced ICAM-1 expression. At the same time, circRasGEF1B regulates the stability of mature ICAM-1 mRNA, suggesting that circRasGEF1B may play an important role in the pathogenesis of acute lung injury caused by sepsis (129). Although some studies have not specifically analyzed the role of circRNA in endothelial barrier disruption caused by ALI, they do reflect that circRNA may contribute to pathogenesis of ALI. It is worth noticing that epigenetic changes in patients with ALI caused by sepsis may lead to abnormal reactions of the immune system and the intensification of inflammatory reactions (131, 132). Epigenetics is based on the chromatin level, involving the regulation of chromatin related molecules such as DNA, noncoding RNA and histone, and plays a vital role in various physiological and pathological processes (133). Overall, research on noncoding RNA in ALI caused by sepsis mainly focuses on miRNA, but there is relatively little research on lncRNA and circRNA, and there is a lack of in-depth functional exploration. The pathogenesis of ALI caused by sepsis can be analyzed from the perspectives of lncRNA and circRNA. In addition, there is currently limited research on drug intervention regarding the involvement of noncoding RNA in the pathogenesis of ALI caused by sepsis, for most of the related studies focus on the effects of drugs on related inflammatory factors and pathways. This suggests that we can study the role of drugs targeting noncoding RNA from the perspective of noncoding RNA participation in order to provide more effective treatment measures for clinical practice. The impact of genetic mechanisms on ALI caused by sepsis is complex, and more research is needed to explore its mechanisms and influencing factors in order to provide more accurate and individualized guidance for clinical treatment.

With the rapid development of intelligent medicine, new discoveries have been made to explore the pathogenesis of acute lung injury caused by sepsis through big data, artificial intelligence, and machine learning. Zheng Y et al. collected data from the Gene Expression Comprehensive Database (GEO) and ArrayExpress databases, and used four machine learning algorithms to identify 52 genes as putative biomarkers before screening out five genes of ARHGDIB, ALDH1A1, TACR3, TREM1 and PI3 for predicting acute lung injury with high accuracy, and screened out small molecule compounds (Curcumin, Tretinoin, Acetaminophen, Estradiol and Dexamethasone) as a potential treatment for acute lung injury caused by sepsis (134). Zhang Z et al. found that miR-335-5p exerts anti-inflammatory effects in sepsis and plays an important role in sepsis mortality through gene-related network analysis (135). Bioinformatics analysis can be performed from nucleic acid and protein sequences to analyze the biological and clinical information of the structure and function expressed in the sequences (136). Big data artificial intelligence technology has been widely used in the exploration of disease biomarkers, discovering potential therapeutic targets for diseases, and providing new ideas for clinical practice.

## 4 Conclusion (prospect)

Sepsis is a systemic inflammatory response that occurs during the process of trauma, infection, burns, and other diseases. It is the developmental basis of multiple organ dysfunction syndrome, and the lung is the most susceptible organ in the multiple organ damage caused by sepsis. The pathogenesis of ALI caused by sepsis is very complex, involving inflammation, coagulation, oxidative stress and genetics. During the onset of ALI caused by sepsis, the release and activation of multiple pro-inflammatory cytokines such as TNF-α, IL-1, IL-2, IL-6, and IL-8, as well as abnormalities in systemic coagulation function can accelerate the development of the disease and cause a cascade reaction of systemic inflammatory, leading to immune dysfunction and worse lung injury, which is not conducive to patients’ prognosis. There has been some progress in the study of the pathogenesis of ALI caused by sepsis in clinical practice, but the research on the deep and specific pathogenesis is not detailed, and a large number of studies are mainly based on animal experiments. The pathogenesis in human still needs further verifications. Therefore, the treatment of ALI caused by sepsis is rather difficult.

The treatment of ALI caused by sepsis is a multi-target, multi-level, and comprehensive treatment process that requires multi-disciplinary cooperation and comprehensive treatment methods. Although there are many therapeutic drugs and methods available, none of them is specific. The innovation of therapeutic drugs and methods is based on a profound understanding of the pathogenesis. Drug development can start from the blocking of signal transduction pathways, apoptosis factors, vascular endothelial contraction factors, and the promotion of growth factors for epithelial cell repair in the pathogenesis. Precision medicine is based on the basic characteristics of diseases and the high precision of drugs, forming high level medical technology based on deep understanding of patients, diseases, and drugs. With the deeper discovery and exploration of the mechanism of ALI caused by sepsis, it is believed that more and more effective therapeutic drugs and methods will emerge in the future, greatly improving the rate of successful treatment and patient survival.

## Author contributions

BS: Writing – original draft. ML: Writing – original draft. JZ: Writing – original draft. HK: Writing – review & editing. HL: Writing – review & editing. FZ: Writing – review & editing.

## Glossary

ALI: Acute lung injury
S-ALI: Sepsis induced acute lung injury
ARDS: Acute respiratory distress syndrome
TLR: Toll like receptor
TNF-α: Tumor necrosis factor-α
IFN-γ: Interferon-γ
IL-1: Interleukin-1
IL-6: Interleukin-6
IL-8: Interleukin-8
IL-10: Interleukin-10
TGF-β: Transforming growth factor-β
MIF: Macrophage inhibitory factor
SIRS: Systemic inflammatory response syndrome
PAF: Platelet activating factor
OFR: Oxygen free radicals
ROS: Reactive oxygen species
SP-A: Surfactant protein A
miRNAs: MicroRNAs
lncRNAs: Long chain non coding RNAs
circRNA: Circular RNA
LPS: Lipopolysaccharide
